# Seed Biopriming for Climate Stress Resilience: Molecular, Physiological, and Epigenetic Mechanisms

**DOI:** 10.3390/ijms27157022

**Published:** 2026-08-05

**Authors:** Iman Janah, Fatima-Ezzahra Soussani, Fatima-Zahra Akensous, Mohamed Ait-El-Mokhtar, Raja Ben-Laouane, Abdelilah Meddich, Marouane Baslam

**Affiliations:** 1 Center of Agrobiotechnology and Bioengineering, Research Unit Labelled CNRST (Centre AgroBiotech-URL-7 CNRST-05), Cadi Ayyad University, Marrakesh 40000, Morocco; 2 Laboratory of Biotechnology, Agri-Food, Materials, and Environment (LBAME), Faculty of Science and Techniques—Mohammedia, Hassan II University of Casablanca, Mohammedia 28800, Morocco; 3 FSTE-FSM Joint Laboratory: NRHE-UMI, Bioresources, Environment and Health Research Team, Moulay Ismail University, Meknes 50050, Morocco; 4 GrowSmart, Paul van Vlissingenstraat 10 F, 1096BK Amsterdam, The Netherlands

**Keywords:** seed biopriming, seed–microbiome interactions, stress resilience, metabolomics, proteomics, epigenetic memory, synthetic microbial communities, SynComs, climate change adaptation

## Abstract

The mutualistic association between plants and their seed-associated microbiota has emerged as a key determinant of crop productivity, influencing plant nutrition, immunity, and tolerance to abiotic stress. Seed biopriming, the controlled application of beneficial microorganisms to seeds before sowing, exploits this interaction to enhance germination, seedling establishment, and stress resilience. Unlike conventional chemical priming, seed biopriming induces coordinated molecular reprogramming through changes in the seed metabolome, proteome, and epigenome. This review synthesizes current evidence demonstrating that seed biopriming promotes the accumulation of osmoprotectants, strengthens antioxidant defenses, enhances secondary metabolism, and generates priming-specific proteomic responses. We further examine how these changes interact with phytohormonal signaling networks and epigenetic mechanisms, including DNA methylation, histone modification, and small RNA-mediated regulation, to establish stress memory and improve plant adaptation. The review also discusses recent advances in synthetic microbial communities and nanobiotechnology for improving inoculant stability and efficacy. Despite promising progress, large-scale application remains constrained by inconsistent field performance, formulation stability, and regulatory challenges. Finally, we highlight the integration of multi-omics and artificial intelligence as promising approaches to improve mechanistic understanding, optimize microbial selection, and accelerate the development of reliable seed biopriming strategies for sustainable agriculture under climate change.

## 1. Introduction: The Seed as an Integrative Biological System

The seed marks a critical transition in the life of spermatophytes, where developmental potential meets environmental input and shapes future plant performance [1]. Formed after fertilization, a seed encloses an embryo within nutrient reserves and a protective coat; its role goes well beyond structural protection and dormancy [2]. Seeds sense and integrate multiple environmental cues, including temperature gradients, water potential, light quality, and hormonal levels, and this combined input largely determines when and how germination proceeds [3,4].

Seed priming exploits this capacity: exposing seeds to controlled stimuli before sowing reprograms their physiological and molecular state ahead of germination [5,6,7]. Conventional priming methods rely mainly on chemical treatments (osmotic agents, phytohormones, antioxidants, and mineral nutrients) to improve germination rate and early seedling vigor [8,9,10]. These approaches work well under controlled conditions [5,11,12,13,14,15], but their effects are often short-lived once seeds face real field conditions: fluctuating stress, complex soil microbiota, and unpredictable climate variability. Chemical priming modulates individual physiological pathways, offering short-term improvements in seed performance rather than sustained stress memory or long-term metabolic reprogramming [16]. In contrast, it does not directly exploit the seed-associated microbiome, which is increasingly recognized as an important determinant of plant resilience.

As climate variability intensifies, with increasingly frequent droughts, heat waves, salinity, and the simultaneous occurrence of multiple stresses, the limitations of conventional chemical priming have become more apparent [17,18]. In this context, biopriming has emerged as a promising alternative that exploits natural plant–microbe interactions to enhance seed performance and stress resilience. Initially described as the application of beneficial microorganisms or biologically derived products to seeds [19,20,21,22], the concept has evolved toward a holobiont perspective, in which the seed is viewed as the starting point for the establishment of a coordinated plant–microbiome system.

Within this framework, biopriming operates, in many cases, through interconnected regulatory layers: the early assembly of functional microbiomes [23,24]; the establishment of epigenetic stress memory [25]; and the metabolic pre-loading of protective compounds [21]. By engaging these multiple levels simultaneously, seed biopriming operates as a systems-level intervention, one that aligns seed biology with the dynamic demands of modern agroecosystems. This review dissects the molecular, physiological, and epigenetic mechanisms through which seed biopriming enhances plant stress resilience, discusses the translational challenges in moving from the laboratory to the field, and outlines emerging technological horizons including SynCom design, nanobiotechnology, and AI-driven precision biopriming. Existing reviews on seed treatments typically address microbial inoculation, epigenetic priming, or synthetic community design as separate research areas. This review instead connects these mechanisms within a single framework, following the sequence of events from early microbial perception at the seed surface to epigenetic memory formation and, finally, to the practical challenges of deploying synthetic microbial communities in the field.

## 2. The “Zero Hour” Molecular Dialog

Seed biopriming generally follows four steps. First, the microbial inoculum is prepared by culturing the selected microorganism(s) to a target concentration. Seeds, surface-sterilized when necessary, are then soaked or coated in this suspension for 2 to 24 h. The treated seeds are subsequently re-dried to their original moisture level to stop germination and keep microbes alive. Finally, the bioprimed seeds are either sown immediately or stored under controlled temperature and humidity until planting, as storage conditions strongly influence microbial survival and subsequent field performance. During the initial soaking or coating phase, the seed molecularly engages with its microbial and soil environment, making this step particularly decisive for the success of subsequent microbial establishment [26]. The first 0–6 h of water uptake are the ‘Zero Hour’, when the seed shifts from dormancy to active signaling. This is the moment at which seed exudates first reach the soil matrix, beneficial and pathogenic microorganisms begin to be recruited or excluded, and the earliest hormonal and redox signals are generated, well before germination itself becomes visible.

### 2.1. The Spermosphere as an Early Signaling Arena

Upon imbibition, seeds immediately interact with the soil environment [27]. This zone, the spermosphere, forms as seeds release carbon-rich exudates into the soil [28]. Here, soil microbes interact, shaped by soil properties and the chemical specificity of seed-derived signals [29]. The seed actively selects and structures this early microbiome, providing competitive advantages against pathogens and environmental stressors [30].

During the first 0–6 h of water uptake, seeds release low-molecular-weight molecules like amino acids, sugars, and organic acids [31]. These exudates act as molecular beacons driving microbial chemotaxis and enabling rapid colonization by specific microbial taxa [30]. Exudate composition changes during germination to regulate the microbiome over time [32]. A well-characterized example is provided by bean seed exudates: malic acid has been shown to stimulate the growth, biofilm formation, and expression of biofilm-related genes (*TasA*, *EpsD*, and *Spo0A*) in *Bacillus amyloliquefaciens*, thereby facilitating early colonization of the seed surface [33]. This early association enhances microbial persistence, improving seedling growth and stress tolerance [33].

### 2.2. Beneficial Microorganisms and Their Mechanistic Contributions

Seed biopriming uses beneficial microbes such as *Trichoderma* spp., *Pseudomonas* spp., *Bacillus* spp., arbuscular mycorrhizal fungi (AMF; *Glomus* spp.), and plant growth-promoting diazotrophs such as *Azospirillum*, *Azotobacter*, and *Rhizobium* [33,34,35,36,37,38]. While promising, their efficacy is strain- and context-dependent. Strains vary from promoting growth to reducing performance based on host and environmental compatibility (Table 1). They function via several mechanisms: nitrogen fixation, phosphorus solubilization, siderophore production, 1-aminocyclopropane-1-carboxylate (ACC) deaminase activity, antifungal compound secretion, and metabolite biosynthesis [38,39,40]. Their collective impact encompasses nutrient mobilization, root architecture modulation, systemic resistance induction, redox homeostasis regulation, and suppression of phytopathogens via antibiosis and mycolytic activity [41,42,43].

### 2.3. Phytohormonal Pre-Shunting by Seed-Colonizing Microorganisms

**Figure 1:**
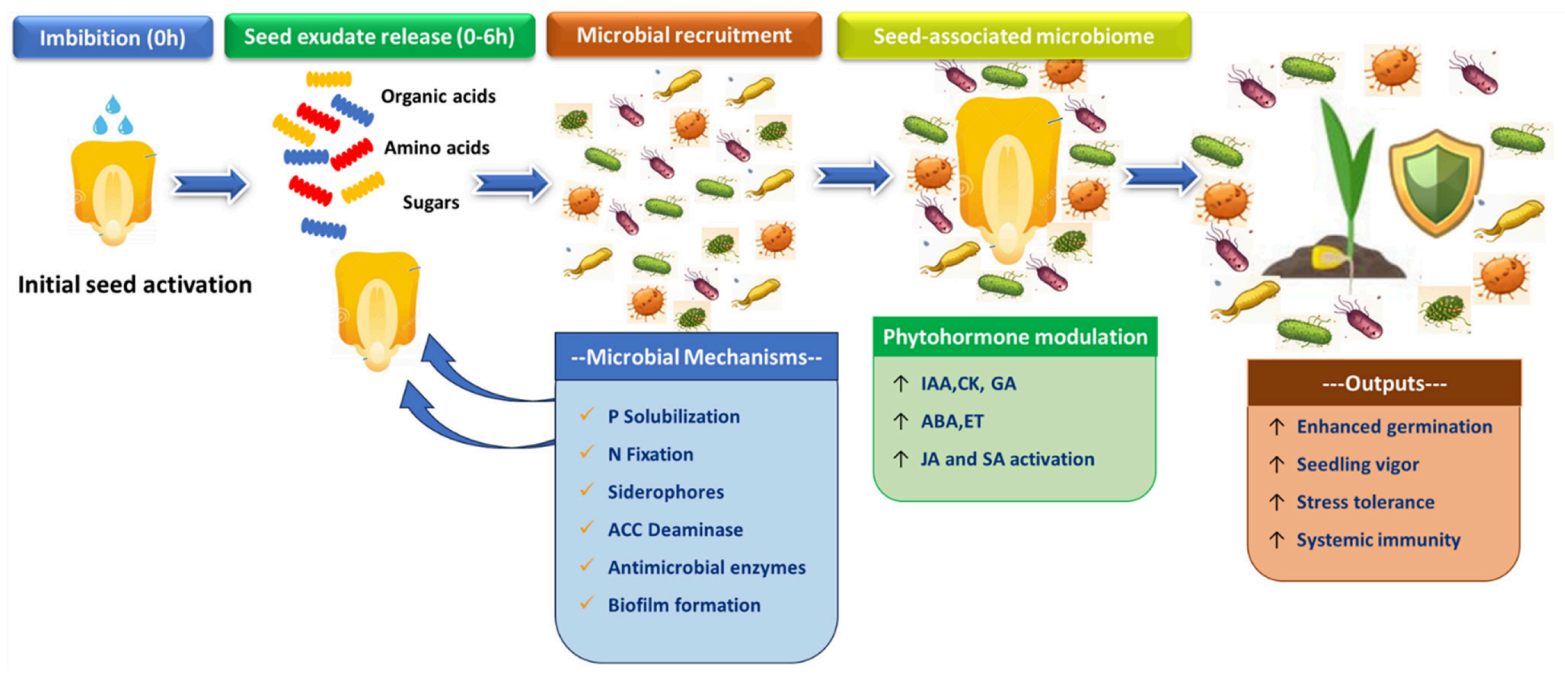
‘Zero-Hour’ molecular dialog during seed biopriming. Schematic representation of the spermosphere interactions and phytohormonal signaling cascades initiated during early imbibition. ABA, abscisic acid; ACC, 1−aminocyclopropane−1−carboxylate; CK, cytokinin; ET, ethylene; GA, gibberellic acid; IAA, auxin; JA, jasmonic acid; N, nitrogen; P, phosphorus; SA, salicylic acid. This figure was created using Microsoft PowerPoint.

Beneficial microbes precondition hormonal networks by modulating defense-related jasmonates (JA) and salicylates (SA), growth-promoting auxins (IAA), cytokinins (CKs), and gibberellins (GA), as well as stress-associated abscisic acid (ABA) and ethylene (ET) [52,53] (Figure 1). Under stress conditions, *Azospirillum brasilense* and several *Bacillus* species modulate ABA, ET, GA, and IAA to improve growth and stress tolerance in maize, soybean, wheat, and tomato [54,55,56,57]. Similarly, *Pseudomonas*, *Bacillus*, *Glutamicibacter*, and *Rhizobium* augment IAA production to confer stress resilience in diverse crops [54,55,56,57,58,59,60,61,62].

**Figure 2:**
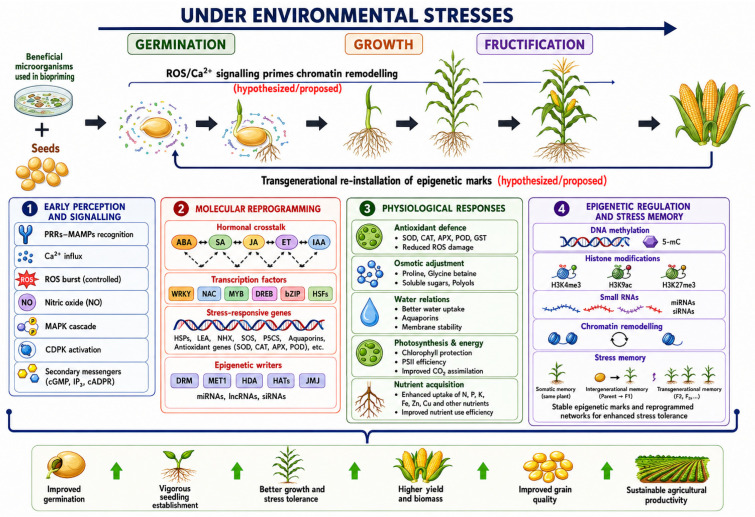
Hypothetical integrated model of the molecular, physiological, and epigenetic pathways activated during seed biopriming under environmental stresses. APX: ascorbate peroxidase; Ca^2+^: calcium ion; cADPR: cyclic ADP-ribose; CAT: catalase; cGMP: cyclic guanosine monophosphate; Cu: copper; Fe: iron; GST: glutathione S-transferase; IP_3_: inositol 1,4,5-trisphosphate; K: potassium; MAMP: microbe-associated molecular pattern; N: nitrogen; NO: nitric oxide; P: phosphorus; POD: peroxidase; PRR: pattern recognition receptors; PSI: photosystem I; ROS: reactive oxygen species; SOD: superoxide dismutase; Zn: zinc. Figure partially created in BioRender. Janah, I. (2026) [https://BioRender.com/qn3f1y6] (accessed on 8 July 2026).

Under abiotic stress, seed mycopriming has been shown to upregulate JA biosynthesis genes in maize, driving accumulation of JA, cis-12-oxo-phytodienoic acid (cis-OPDA), and jasmonoyl-isoleucine (JA-Ile), which in turn activate a cascade of JA-responsive genes, including *lipoxygenase*, *allene oxide synthase*, *12-oxophytodienoate reductase*, and the bHLH transcription factor MYC2 [55]. This JA-dependent transcriptional program reinforces cell wall integrity, stimulates secondary metabolism, and enhances tolerance to osmotic and oxidative stresses (Figure 2).

### 2.4. ROS and RNS Operate as Key Regulatory Nodes

Biopriming also triggers the controlled generation of reactive oxygen species (ROS) and reactive nitrogen species (RNS) during the biopriming window. Rather than being mere by-products, these molecules act as signaling mediators that regulate germination kinetics, metabolism, and stress readiness [63,64,65,66,67]. At low concentrations, ROS and RNS act as signaling molecules that control plant growth and development, symbiotic associations, and defense mechanisms in response to biotic and abiotic stress, whereas excessive accumulation causes cellular damage and growth retardation [68]. However, their role in plant–microbe interactions and biopriming remains largely inferential [69]. These redox signals link microbial cues to downstream hormonal pathways, coordinating transcriptional and physiological adjustments. Thus, the microbe-modulated balance between ROS generation and antioxidant buffering determines priming efficacy and seedling vigor.

## 3. Epigenetic Mechanisms Underlying Stress Resilience

### 3.1. Chromatin Remodeling and DNA Methylation

Biopriming induces long-lasting epigenetic modifications via DNA methylation, histone modifications, and small RNA (sRNA) pathways to enhance stress tolerance [70,71,72]. These mechanisms are central to both the transcriptional and post-transcriptional regulation of stress-response genes through dynamic remodeling of chromatin architecture [73]. These marks can persist across developmental stages or be transmitted to offspring as a heritable stress memory [74].

Biopriming reliably reprograms the seed epigenome [75]. Lephatsi et al. [76] demonstrated that biopriming with a *Bacillus*-based consortium maintained higher global DNA methylation under drought stress than non-inoculated controls. A comparable mechanism has been reported in *Brachypodium distachyon*, where colonization by *Bacillus subtilis* B26 upregulated the DNA methyltransferases *MET1B*-like, *CMT3*-like, and *DRM2*-like, driving genome-wide hypermethylation that stabilized expression of the drought-responsive genes *DREB2B*-like, *DHN3*-like, and *LEA-14-A*-like under water deficit [77]. In parallel, increased histone acetylation, associated with chromatin opening and transcriptional activation, has been linked to biopriming-induced upregulation of growth-promotion and stress-resilience genes, ultimately facilitating improved germination and seedling establishment under adverse conditions [25,78].

### 3.2. Small RNA-Mediated Cross-Kingdom Regulation

Endogenous sRNAs regulate plant stress memory and can be modulated by biopriming signals. Perception of these microbial signals can activate the plant RNA interference (RNAi) machinery, leading to the selective silencing of susceptibility genes and the fine-tuning of stress-responsive pathways, a mechanism demonstrated primarily in root and leaf interactions rather than in bioprimed seeds themselves [79,80,81]. In Arabidopsis, for instance, expression of a hairpin RNA directed against bacterial virulence genes reduces stomatal reopening and slows pathogen growth, an effect that disappears in *dcl2*/*dcl3*/*dcl4* mutants, confirming the direct involvement of the plant’s RNAi machinery [82]. In tomato, endogenous miRNA expression is altered in response to colonization by *Trichoderma asperellum*, targeting genes involved in ET and oxidative stress responses [83]. These two examples are drawn from root interaction systems and are presented as mechanistic precedent rather than as demonstrated evidence within bioprimed seed tissue itself; whether comparable uptake, stability, and RNAi-dependent silencing occur during the seed biopriming window specifically remains an open and largely untested question. Microbially triggered sRNAs could bridge initial microbial recognition and long-lasting molecular memory by repressing negative regulators of stress tolerance. This RNA-based regulation complements DNA and histone modifications, reinforcing the multi-layered molecular program installed by biopriming [79,80,81].

### 3.3. Transcriptomic Evidence for Persistent Priming Memory

Recent transcriptomic evidence confirms that biopriming establishes persistent transcriptional memory. RNA-seq profiling of *Silene sendtneri* seeds bioprimed with *Paraburkholderia phytofirmans* revealed a distinct transcriptomic state prior to stress exposure, characterized by enrichment of genes involved in redox homeostasis, metal ion detoxification, ROS signaling, cell wall remodeling, and phenylpropanoid biosynthesis [25].

Bioprimed seeds also show pre-activation of a coordinated detoxification and signaling network, including chloroplastic glutathione reductase, RBOH (respiratory burst oxidase homolog)-like NADPH (nicotinamide adenine dinucleotide phosphate) oxidases, class III peroxidases, and multiple WRKY transcription factors (Figure 2). This state allows controlled ROS signaling and faster redox buffering to maintain homeostasis. Concurrently, induction of delta-1-pyrroline-5-carboxylate synthetase and glycosyltransferases shifts metabolism toward proline accumulation, osmoprotection, sugar synthesis, and cell wall remodeling [25]. For ion transport, upregulation of ABC (ATP-binding cassette) transporters, CAX2 (cation/H^+^ exchanger 2)-like vacuolar exchangers, and zinc/metal transporter families may enhance ion detoxification and redistribution, while selective repression of NRAMP (natural resistance-associated macrophage protein)- and Mg^2+^ transporter homologs may stabilize nutrient balance during early germination [25].

### 3.4. Intergenerational and Transgenerational Priming Memory

**Figure 3:**
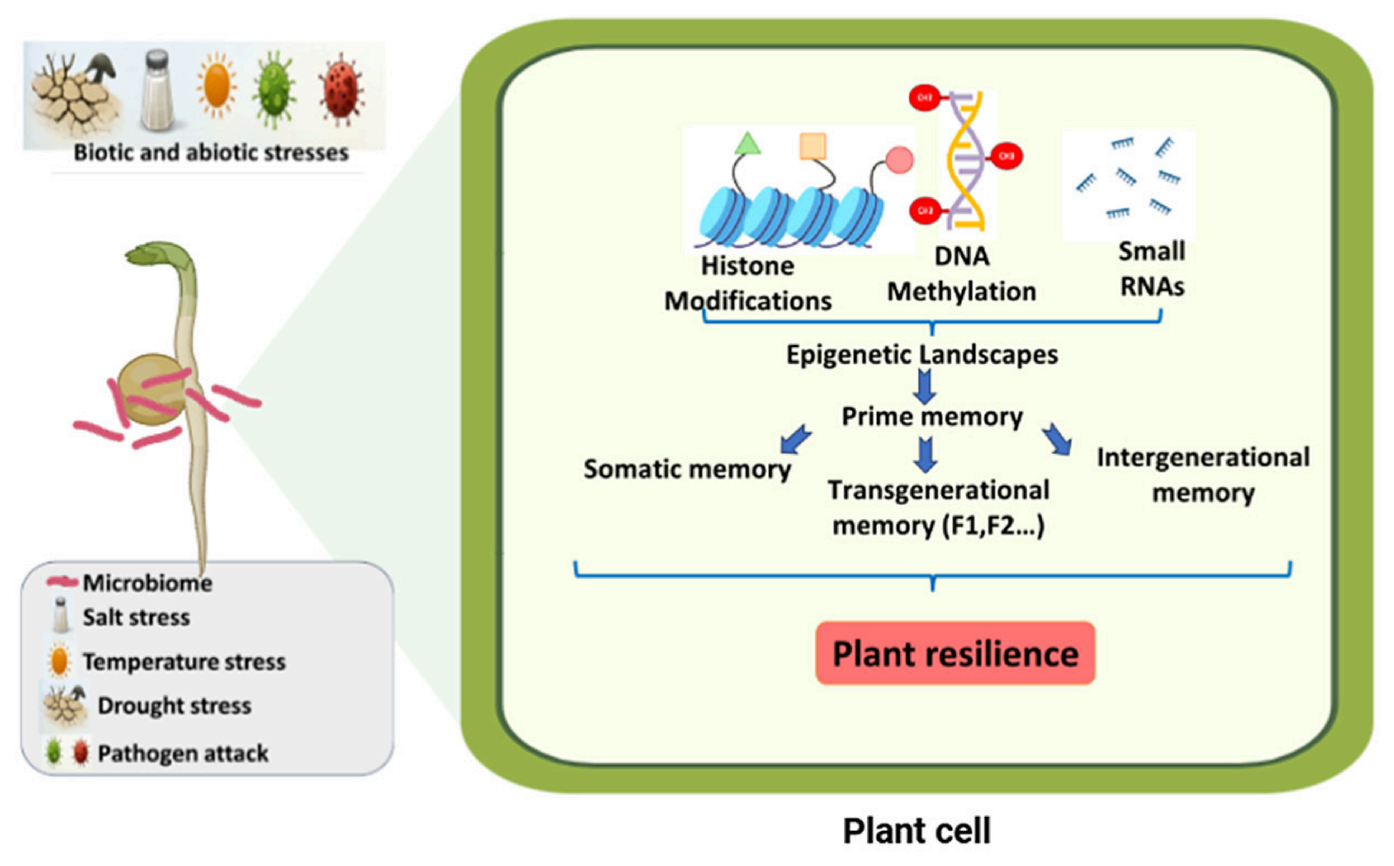
Conceptual overview of epigenetic stress memory establishment in bioprimed seedlings. Seed-associated microbiome components interact with the germinating seedling under biotic and abiotic stresses. This interaction triggers epigenetic landscape remodeling, involving histone modifications, DNA methylation, and small RNA (sRNA)-mediated regulation, collectively referred to as the “epigenetic landscape”. These changes converge into a “prime memory” state, which can be propagated as somatic memory (within the same plant), transgenerational memory (stably inherited across F1, F2, and subsequent generations), or intergenerational memory (transmitted to the immediate offspring generation). These layers of epigenetic memory contribute to enhanced plant resilience against recurring or novel environmental stresses. This schematic illustrates the conceptual relationships between these processes rather than their detailed molecular mechanisms, which are discussed in Section 3.3 and Section 3.4. Figure partially created in BioRender. Janah, I. (2026) [https://BioRender.com/1pi75cz] (accessed on 1 June 2026).

Beyond somatic memory, biopriming may also establish intergenerational and transgenerational epigenetic memory, passing stress resilience to subsequent generations. However, these transgenerational effects lack extensive experimental validation [84]. For example, in tomato, biopriming with *Trichoderma harzianum* has been shown to modulate DNA methylation and histone acetyltransferase, perpetuating stress memory and enhancing resilience across generations [85]. In barley, *Trichoderma*-mediated priming under drought stress not only enhanced tolerance and yield in the treated generation but also protected the subsequent generation [86]. This transgenerational effect was associated with elevated expression of the epigenetic regulator *HvDME* (*Hordeum vulgare DEMETER*), a DNA glycosylase involved in active DNA demethylation, independently characterized as drought-responsive and differentially methylated across barley cultivars [86,87]. These findings point to a “prime memory” state branching into somatic, transgenerational, and intergenerational plant resilience. Figure 3 summarizes how these DNA methylation and histone modification changes, together with the sRNA-mediated mechanisms discussed above, fit within this broader epigenetic landscape established during biopriming, and how this landscape can be propagated as somatic, intergenerational, or transgenerational memory. This framework remains largely descriptive; the mechanisms, durations, and boundary conditions of inter/transgenerational priming are still poorly defined and represent a priority for future research.

### 3.5. An Integrated Hypothetical Model of Biopriming-Induced Pathways

The proposed life-cycle cascade (Figure 2) comprises four interconnected stages: (1) early perception and signaling, (2) molecular reprogramming, (3) physiological responses, and (4) epigenetic regulation and stress memory. Beneficial microorganisms used in biopriming interact with seeds to trigger early perception and signaling: PRR (pattern recognition receptors)-MAMP (microbe-associated molecular patterns) recognition, Ca^2+^ influx, controlled ROS burst, nitric oxide, MAPK (Mitogen-Activated Protein Kinase) cascade, CDPK (Calcium-Dependent Protein Kinase) activation, and secondary messengers including cyclic guanosine monophosphate (cGMP), inositol 1,4,5-trisphosphate (IP_3_), and cyclic ADP-ribose (cADPR). Among these early signals, calcium and ROS in particular have been mechanistically linked to downstream chromatin remodeling, and we propose that this redox–calcium axis may initiate epigenetic reprogramming, thereby connecting early perception events to the epigenetic regulatory layer described below.

This link is supported by direct experimental evidence, though not yet demonstrated within a biopriming system specifically: in Arabidopsis, the ROS-responsive DNA demethylase ROS1 actively demethylates the DOGL4 promoter to regulate seed dormancy and ABA sensitivity [88], and *ros1* mutants show DNA hypermethylation and reduced expression at ABA-inducible loci during early seedling development [89]. Genome-wide DNA hypomethylation has similarly been observed in H_2_O_2_-overproducing transgenic tobacco, correlating with enhanced stress resistance [90]. Upstream of this redox–epigenetic link, cytosolic calcium elevation has been shown to be required for RBOH-mediated apoplastic ROS production in Arabidopsis leaves [91]. In parallel, molecular reprogramming integrates hormonal crosstalk, activation of stress-responsive transcription factors, and induction of stress-responsive genes. This transcriptional and metabolic pre-loading is directly supported by RNA-seq profiling of *Silene sendtneri* seeds bioprimed with *Paraburkholderia phytofirmans*, previously mentioned, which revealed a distinct transcriptomic state enriched in redox homeostasis, metal detoxification, and ROS-signaling genes, established at the seed stage prior to any cadmium exposure [25]; notably, the same strain also induces long-term metabolic and transcriptional changes underlying salt tolerance in *Arabidopsis thaliana* [72], suggesting that this reprogramming generalizes across host species and stress types. In maize, biopriming with *Bacillus nematocida* combined with drought stress upregulated the stress-responsive genes *PLD*, *PYL1*, *SLAH1*, and *OST1* by up to 60-, 63-, 11-, and 7-fold, respectively [35]. A comparable pattern extends to biotic stress: soybean seeds coated with *Bacillus simplex* Sneb545 showed integrated transcriptomic and metabolomic reprogramming that elevated nematicidal metabolites under nematode infection [92]. These events trigger physiological shifts in antioxidant defense, osmotic adjustment, photosynthesis, and nutrient acquisition. Concurrently, epigenetic regulation, comprising DNA methylation, histone modifications (*H3K4me3*, *H3K9ac*, *H3K27me3*), small RNA activity, and chromatin remodeling, establishes a stable stress memory. Among these mechanisms, sRNA involvement has already been demonstrated in seed biopriming, as *Bacillus licheniformis*-bioprimed maize seeds showed *miR160d* upregulation under salt stress, together with enhanced antioxidant enzyme activities and improved seedling growth [93]. A feedback loop indicates that this epigenetic memory is proposed to be transgenerationally re-installed in progeny seeds, closing the cycle back to the initial biopriming step. This is consistent with persistent *HvDME* expression documented across barley generations following *Trichoderma*-mediated priming [86,87]. These integrated molecular, physiological, and epigenetic pathways are hypothesized to translate into improved germination, vigorous seedling establishment, better growth and stress tolerance, higher yield and biomass, improved grain quality, and, ultimately, sustainable agricultural productivity. This scheme represents a literature-based working hypothesis rather than an experimentally validated pathway map. While some of the individual mechanisms described above have been experimentally demonstrated in plants, others remain inferred from related biological systems. Their integration into a unified signaling cascade during seed biopriming, particularly the link between ROS/RNS signaling and epigenetic reprogramming at the seed stage, has not yet been experimentally demonstrated and should be considered a hypothetical model requiring future multi-omics validation.

## 4. From Single Strains to Synthetic Communities

### 4.1. Limitations of Monoculture-Based Biopriming

Single-strain inoculants have clear limits once biopriming moves from lab conditions to real field environments. They lack the diversity, resilience, and adaptability to handle the simultaneous stresses crops encounter in natural soils [94]. Soil heterogeneity, competition from native microbiota, climatic variability, and host genotype effects frequently suppress the activity of inoculated strains, limiting their long-term efficacy [95,96]. Furthermore, a single strain cannot address simultaneous osmotic, oxidative, nutritional, and hormonal disruptions [97].

A few examples illustrate both the value and the limits of single-strain approaches. Biopriming tomato seeds with *Bacillus aryabhattai* can suppress *Fusarium* wilt [98], and applying *B. licheniformis* to maize mitigates salinity by modulating antioxidant enzyme activities and miRNA expression [93]. Similarly, wheat seeds bioprimed with drought-tolerant *T. harzianum* strains alleviates water deficiency stress via antioxidant defense mechanisms [99]. However, these benefits remain stress-specific, cultivar-dependent, and environmentally contingent.

### 4.2. Rational Design and Ecological Principles of SynComs

**Figure 4:**
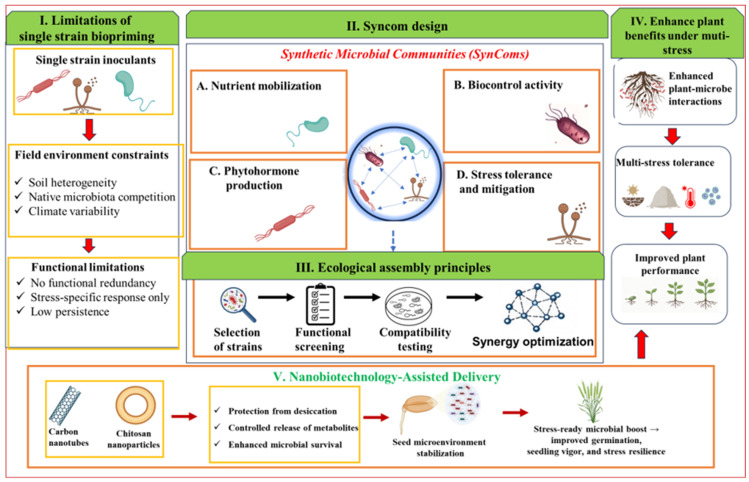
From single-strain inoculants to engineered synthetic microbial communities (SynComs) for next-generation seed biopriming. (**I**) Single-strain inoculants often show inconsistent field performance due to soil heterogeneity, competition with native microbiota, climate variability, and limited functional redundancy. (**II**) SynComs combine microorganisms with complementary traits, including nutrient mobilization, phytohormone production, and biocontrol activity, providing several plant growth-promoting functions simultaneously. (**III**) Rational SynCom assembly follows sequential ecological steps: strain selection, functional screening, compatibility testing, and synergy optimization. (**IV**) Well-designed SynComs strengthen plant–microbe interactions and improve tolerance to multiple, potentially combined, stresses. (**V**) Nanobiotechnology-assisted delivery (e.g., carbon nanotubes, chitosan nanoparticles) protects inoculants from desiccation, enables controlled metabolite release, and improves microbial survival, seed germination, and seedling vigor. SynComs: synthetic microbial communities. Figure partially created in BioRender. Janah, I. (2026) [https://BioRender.com/332osm2] (accessed on 1 June 2026).

Synthetic microbial communities (SynComs), defined consortia of selected microbial species assembled based on functional complementarity, represent a conceptual and practical advance beyond single-strain inoculants [100]. SynComs are designed to collectively deliver specific functional traits relevant to plant health, including secondary metabolite production, biofilm formation, and systemic resistance induction. By combining strains with synergistic functions, SynComs mimic natural microbiomes, stabilize plant–microbe interactions, and enhance multi-stress tolerance [101] (Figure 4). A four-member SynCom comprising *Arthrobacter*, *Brevibacterium*, *Enterobacter*, and *Plantibacter*, with complementary traits like phosphorus solubilization, IAA production, and *Fusarium oxysporum* biocontrol, outperformed individual strains in promoting cotton growth [102]. Similarly, specific SynCom combinations derived from wheat rhizosphere bacteria outperformed single strains in suppressing *Rhizoctonia solani*-induced root rot [103]. In *Medicago sativa*, SynComs composed of *Ensifer* and *Pseudomonas* strains significantly enhanced plant growth, nodulation, and photosynthesis under combined drought, salinity, metal contamination, and heat stress in estuarine soils via antioxidant defense activation [104]. In *Zea mays*, bacterial SynComs orchestrated coordinated above- and below-ground responses under drought and phosphorus deficiency, enhancing rhizosheath formation, water retention, root colonization, and biomass accumulation [105]. Table 2 summarizes SynCom compositions evaluated in biopriming studies.

### 4.3. Nanobiotechnology for SynCom Stabilization

Recent advances in nanobiotechnology are opening new avenues for stabilizing and delivering SynComs during seed biopriming [112]. Carbon-based nanomaterials, including carbon nanotubes [113], and biopolymers such as chitosan nanoparticles [114] can serve as protective nano-scaffolds for beneficial microorganisms, shielding them from desiccation, oxidative stress, and nutrient fluctuation [115,116]. These nanocarriers also enable the sustained release of microbial metabolites, phytohormones, and stress-modulating compounds into the seed microenvironment [94,117,118]. This synergy amplifies SynCom efficiency, improving germination, seedling vigor, and stress resilience [112,119,120,121].

Despite these advantages, nanobiotechnology-assisted biopriming also carries practical and biosafety limitations. Nanoparticle effects are dose-dependent: low concentrations can prime defense responses, but higher doses cause phytotoxicity, oxidative damage, and reduced chlorophyll content [122]. Their environmental fate is poorly understood, as soil persistence and multi-season impacts on non-target microbes and SynCom strains are rarely studied [122,123].

Current toxicity testing also relies mainly on short-term laboratory assays, which do not capture chronic or long-term effects [123]. Overall, nanobiotechnology-assisted biopriming remains promising but requires more field-based, long-term safety data before it can be recommended for large-scale use [122,123].

## 5. Multi-Omics Integration of Metabolic Reprogramming

### 5.1. Metabolomics and Primary Metabolic Reprogramming

Metabolomics captures the downstream outputs of the genome, transcriptome, and proteome to reflect physiological states [124,125,126]. This early reprogramming simultaneously affects energy production, carbohydrate mobilization, amino acid metabolism, and secondary metabolite pathways [5]. For instance, metabolomic profiling of wheat seeds bioprimed with the PGPR strains *Paenibacillus alvei* (T22) and *Bacillus subtilis* revealed differential accumulation of phenylpropanoids, organic acids, lipids, and benzenoid compounds in both rhizosphere and leaf tissue, indicating that seed-level bio-priming triggers systemic metabolic changes [127]. These latent, mobilizable metabolites lower the energetic cost of defense by eliminating the need for de novo synthesis during stress.

Metabolomic profiling has demonstrated that biopriming modulates key antioxidant enzymes, including superoxide dismutase (SOD), catalase (CAT), and ascorbate peroxidase (APX), that reduce ROS accumulation and mitigate oxidative damage under stress [128,129]. Biopriming also alters primary metabolites like amino acids, organic acids, and fatty acids to maintain energy homeostasis [130,131,132]. Secondary metabolites, particularly flavonoids and phenolics, consistently increase after biopriming and contribute to both abiotic stress tolerance and pathogen resistance [133]. For example, in cumin, biopriming with *Trichoderma harzianum* activated the phenylpropanoid pathway and boosted essential oil constituents (cuminaldehyde, γ-terpinene, p-cymene, β-pinene), shifting the plant’s metabolome toward a defense-ready state before any stress exposure [134]. Osmoprotectants (proline, glycine betaine, polyamines) and antimicrobial compounds follow the same accumulative pattern [135,136].

### 5.2. Proteomic Signatures of Biopriming

**Figure 5:**
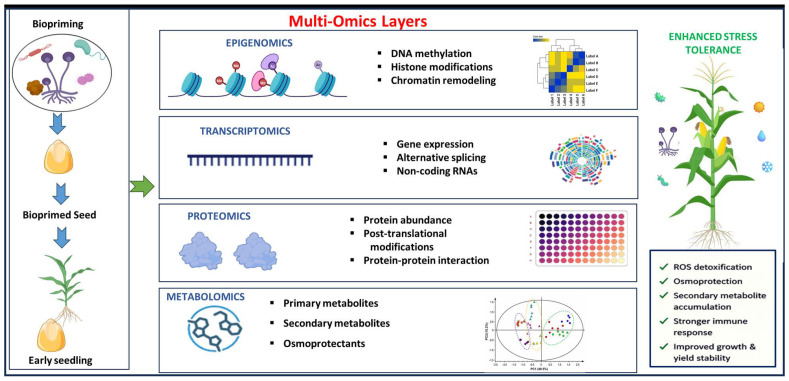
Multi−omics integration framework in seed biopriming for enhanced stress tolerance. Biopriming inoculum is applied to the seed, which develops into a bioprimed seed and subsequently an early seedling (left panel). This process reprograms four interconnected omics layers (center): epigenomics (DNA methylation, histone modifications, chromatin remodeling), transcriptomics (gene expression, alternative splicing, non−coding RNAs), proteomics (protein abundance, post−translational modifications, protein−protein interactions), and metabolomics (primary and secondary metabolites, osmoprotectants). Integration of these layers converges on enhanced stress tolerance (right panel), reflected in ROS detoxification, osmoprotection, secondary metabolite accumulation, a stronger immune response, and improved growth and yield stability. Figure partially created in BioRender. Janah, I. (2026) [https://BioRender.com/0hk79l9] (accessed on 1 June 2026).

Biopriming also leaves a measurable imprint at the proteome level. According to Bhaskaran et al. [137], the proteomic analysis of bioprimed seeds and seedlings identified distinct protein signatures that clearly separated bioprimed plants from hydro-primed controls. These signatures feature an increased abundance of energy production and carbon metabolism proteins to fuel rapid germination [137]. Biopriming also increases stress-related, redox-regulatory, and detoxification proteins, creating a pre-activated protective state [137]. Thus, multi-omic evidence confirms that biopriming establishes a pre-conditioned state that accelerates defense activation (Figure 5).

### 5.3. Towards Multi-Omics Integration

Because no single approach captures biopriming dynamics, integrating transcriptomics, metabolomics, proteomics, and epigenomics is essential to identify regulatory hubs, rate-limiting steps, and biomarkers that predict field performance [71,138] (Figure 5). In cherry, for example, combined phytohormone metabolomics and transcriptomics across three PGPR strains identified strain-specific co-expression modules linked to hormone regulation and cell wall remodeling, showing a temporal shift from early metabolic priming to later structural growth [139]. Machine learning applied to multi-omics datasets may help identify genotype × microbiome × environment interactions and guide microbiome-based interventions suited to specific agroecological contexts.

## 6. Translational Challenges and Critical Bottlenecks

### 6.1. Laboratory-to-Field Translation Gap

The translation gap between controlled environments and field settings is compounded by inconsistent methodological reporting across the SynCom literature [140,141]: key parameters such as inoculation method, number of validation sites, and direct comparison against individual constituent strains are frequently not reported in published studies, limiting comparative evaluation of SynCom efficacy and complicating meta-analytic synthesis across the field. Laboratory-documented successes (i.e., optimal germination rates and enhanced seedling vigor) frequently fail to translate reliably to the field, where environmental conditions are dynamic, complex, and unpredictable [142]. Field efficacy is attenuated by poor inoculant survival, native microbial antagonism, altered root development, and concurrent abiotic stresses [143,144]. Furthermore, the longevity of these post-transplantation effects remains a major knowledge gap.

### 6.2. Formulation, Viability, and Storage

Commercial scaling up of seed biopriming introduces technical challenges regarding microbial viability, formulation stability, and storage (Table 3). Drying temperatures, ambient fluctuations, and prolonged storage reduce microbial survival [145,146]. This trade-off is illustrated by a recent study in which microencapsulation of a wheat rhizosphere PGPR strain in potassium alginate–pectin microcapsules maintained approximately 70% cell viability after 28 days of storage, compared with only 42% in the non-encapsulated formulation [147]. In addition, variations in formulation, priming duration, inoculum preparation, and coating materials compromise reproducibility [148]. Delayed sowing after priming also risks reducing seed vigor by accelerating post-priming metabolism during storage [149].

### 6.3. Regulatory and Biosafety Considerations

Applying CRISPR-engineered microorganisms in seed biopriming represents a significant biotechnological opportunity but also raises substantial regulatory and ethical challenges [157,158]. In the EU, CRISPR-Cas microbes are classified as GMOs, requiring stringent safety evaluations and market authorizations that delay adoption [159,160]. International approaches to genome-edited microorganisms vary considerably, fragmenting market access for biopriming technologies based on engineered strains [161]. Concerns also include horizontal gene transfer, environmental impacts, and native microbiome disruption [162]. Overcoming these barriers requires collaboration between scientists, regulatory bodies, and policymakers.

## 7. Future Horizons: Precision Biopriming

### 7.1. AI and Machine Learning Integration

Artificial intelligence (AI) and machine learning (ML) can optimize biopriming inoculant design. Input data include seed microbiome profiles, plant multi-omics, soil properties, and climate variables. Models can predict germination rates, seedling vigor, and colonization, though predicting complex stress resilience remains difficult due to limited datasets. A typical model, such as a random forest or a deep neural network, is trained on these data and tested using cross-validation to check that its predictions hold up on new samples rather than just the ones it was trained on [163,164].

Several problems still limit this approach in seed biopriming specifically. Most available datasets come from controlled greenhouse studies, not multi-season field trials. Differences in sequencing equipment or protocols between labs can create batch effects that look like biological signals but are not, unless corrected for [165]. Furthermore, environmental and cultivar variations reduce model generalizability across new locations. One preprint study illustrates a possible application: AI-based selection combined with a nanoparticle-microbial seed coating improved germination and seedling growth under drought stress [166]. Resolving these gaps requires large, standardized, field-based datasets tailored specifically to seed biopriming.

### 7.2. Synthetic Biology and Microbiome Engineering

Synthetic biology can enhance seed biopriming efficacy and consistency [167,168]. Engineered strains can optimize stress responses, rhizosphere colonization, and growth-promoting metabolite biosynthesis. CRISPR-based genome editing allows precise modification of microbial genes associated with plant-beneficial functions, including ACC deaminase activity, siderophore production, phytohormone biosynthesis, and nutrient mobilization [169,170]. These modifications provide more predictable performance than conventional strain selection.

Nevertheless, the practical application of engineered microorganisms requires careful consideration of ecological fitness, long-term stability, and interactions with native microbial communities. Future research should therefore integrate synthetic biology with microbiome engineering approaches to develop inoculants that maintain functional compatibility with indigenous microbiota while minimizing potential ecological disturbances. In parallel, comprehensive biosafety assessments and compliance with applicable regulatory frameworks will be essential before the agricultural deployment of genetically engineered microbial inoculants.

### 7.3. Climate-Adaptive Seed Formulations

Intensifying climate variability requires biopriming formulations designed for multi-stress resilience rather than single-stress tolerance. This requires a systems-level understanding of how SynCom composition, seed exudate chemistry, and epigenetic pre-programming interact with specific stress combinations, including simultaneous drought, heat, and salinity. Developing ‘stress-anticipatory’ formulations with pre-selected, nano-encapsulated microbial communities represents a key direction for next-generation biopriming. Integrating climate scenario modeling with microbiome functional genomics could enable the design of regionally tailored biopriming solutions.

## 8. Conclusions

Seed biopriming has matured from an empirical technique into a mechanistic, systems-level intervention. The seed–microbiome dialog initiated at imbibition engages phytohormonal networks, ROS/RNS signaling, metabolic pre-loading, and epigenetic reprogramming in a highly coordinated manner that collectively pre-conditions plants for enhanced resilience across diverse stress environments.

The transition from single-strain inoculants to rationally designed SynComs, underpinned by ecological principles and stabilized by nanobiotechnology, represents a major advance in translational viability. Integration of multi-omics datasets with AI-driven analytical frameworks promises to unlock the predictive power needed for genotype- and environment-specific precision biopriming.

Critical challenges remain: closing the laboratory-to-field translation gap, standardizing formulation and storage protocols, and navigating regulatory frameworks for next-generation microbial inoculants. Transgenerational epigenetic inheritance of priming memory, a phenomenon with potentially transformative implications for crop improvement, warrants substantially deeper investigation. Beyond these points, three additional limitations currently constrain the field. First, the causal links between the molecular layers described here, particularly the proposed connections between ROS/RNS signaling and epigenetic remodeling and between transgenerational epigenetic marks and their re-establishment in progeny, remain hypothetical and have not yet been demonstrated within a single biopriming system. Second, mechanistic evidence for small RNA-mediated regulation has so far been generated primarily in root and leaf interactions, leaving its relevance to the seed stage itself largely unexplored. Third, most SynCom and multi-omics studies to date remain limited to single-stress, single-crop systems, whereas field conditions typically involve combined and sequential stresses. Addressing these gaps will require direct multi-omics profiling of bioprimed seeds across the priming-to-germination window to test the integrated model proposed here; functional validation of small RNA-mediated silencing specifically in seed tissues using loss-of-function RNAi mutants; longitudinal, multi-generation field trials to determine the durability and heritability boundaries of transgenerational priming memory; and standardized SynCom formulation and storage protocols validated across multiple agroecological contexts to close the gap between controlled-condition efficacy and field-level reproducibility. By converging synthetic biology, multi-omics, and precision agronomy, biopriming can secure sustainable, climate-resilient crop production.

## Figures and Tables

**Table 1:** Effectiveness of the main microbial species used for seed biopriming.

Genus/Species	Crop	Reported Effect	Efficacy	Ref.
*Azotobacter chroococcum* ± AMF	Winter wheat	Increased spikes per m^2^, kernel weight, and grain yield, and enhanced grain protein content by 13% compared with the non-inoculated control	Effective	[44]
*Azospirillum lipoferum* CRT1	Maize	Hastened radicle emergence (6–8 h), increased bacterial colonization, altered primary metabolism, and improved photosynthetic yield and root surface area	Cultivar-dependent	[45]
*Azotobacter chroococcum* + *Azospirillum lipoferum*	Barley	Improved growth, yield, and photosynthetic dry matter remobilization compared to non-inoculated controls	Effective	[46]
*Bacillus licheniformis*/*Enterobacter asburiae*	Quinoa	Improved germination, seedling growth, salinity tolerance, chlorophyll index, and P/K uptake, and reduced Na^+^ accumulation	Highly effective	[47]
*Bacillus* spp., *B. megatherium*, *Azotobacter chroococcum*, *Pseudomonas fluorescens*	Field dodder	Variable effects on germination depending on the strain (stimulatory or inhibitory)	Species-dependent	[48]
*Paraburkholderia phytofirmans* PsJN	Micro-Tom Tomato	Improved fruit yield, Ni stress tolerance, and chlorophyll a and total chlorophyll content, stabilized proline levels, and modulated antioxidant enzyme activity, particularly under severe Ni stress	Effective	[49]
*Enterobacter hormaechei*	Okra	Improved germination parameters, seedling vigor index, plant growth, P and K uptake, leaf surface area, SPAD chlorophyll index, and IAA and siderophore production	Highly effective	[50]
AMF (*Glomus viscosum*)	Artichoke	Enhanced antioxidant defense against *Verticillium dahliae*: increased APX, MDHAR, and SOD activities, higher ascorbate and glutathione content, reduced lipid peroxidation and H_2_O_2_ levels	Effective (mitigates pathogenicity, alleviates oxidative stress)	[51]

AMF: arbuscular mycorrhizal fungi; APX: ascorbate peroxidase; IAA: indole-3-acetic acid; K: potassium; MDHAR: monodehydroascorbate reductase; Na: sodium; Ni: nickel; P: phosphorus; SOD: superoxide dismutase; SPAD: soil–plant analysis development.

**Table 2:** Synthetic microbial communities used in seed biopriming.

SynCom/Microbial Composition	Crop	Stress	Observed Plant Response	Inoculation Method	Validation Environment	Ref.
PGPR consortium: *Ensifer adhaerens* BK-30, *Pseudomonas fluorescens* SN5, *Bacillus megaterium* SN15	Wheat (*T. aestivum*)	Salinity	Increased growth, yield, and stress tolerance	Jar trial + pot trial	Laboratory/greenhouse	[106]
Two-member SynCom: *Chryseobacterium indologenes* ICKM4 and *Stenotrophomonas maltophilia* ICKM15	Chickpea (*C. arietinum*)	Salinity	Improved biochemical, histochemical, and genomic stress responses	In planta evaluation (pot trial)	Laboratory/greenhouse	[107]
Mangrove endophyte SynCom: *Isoptericola* sp. AK164 and *Tritonibacter mobilis* AK171	Rice (*O. sativa*)	Salinity	Shortened life cycle and enhanced yield and salt tolerance	Individual-strain inoculation and SynCom combination, hydroponic and soil conditions	Laboratory	[108]
Stable 15-member bacterial SynCom from stiff brome	*Brachypodium distachyon*	Drought	Improved growth and physiological performance under water deficit	Seed priming	Greenhouse	[109]
Seed-borne SynCom from *Medicago laciniata* and *M. littoralis* (*Pantoea agglomerans*, *P. allii*, *Pseudomonas graminis* DSM)	Alfalfa (*M. sativa*)	Drought	Enhanced germination, seedling establishment, and early growth; host microbiome restructuring	Seed priming	Greenhouse	[110]
*Streptomyces araujoniae* consortium (TN11 + TN19)	Chickpea (*C. arietinum*)	*Fusarium* wilt	Improved antioxidant defense, proline levels, and electrolyte homeostasis; strong antifungal metabolites (valinomycin, dinactin, erucamide)	Seed priming	Greenhouse	[111]

**Table 3:** Case studies illustrating key seed biopriming trends and translational challenges.

Plant Species	Biopriming Feature	Experimental Outcomes	Translation Bottlenecks	Ref.
*Phaseolus vulgaris*	*Rhizobium tropici* + silk coating solution (+trehalose)	Improved germination and seedling growth under salinity; higher root density than freshly bioprimed seeds; stabilization of physiological traits	High concentrations of priming agents may leach after irrigation; coating formulation is dependent on stress conditions	[150]
*Brassica rapa*	*Trichoderma asperellum* in sodium alginate biopolymer	High spore viability; sodium alginate showed superior carrier performance compared with alternative polymer	Excessive spore loading inhibited radicle elongation; compatibility between carrier and seed species requires further validation	[151]
*Zea mays*	*Beauveria bassiana*-*T. asperellum* consortium	Enhanced antioxidant enzyme activity, hormone signaling, and defense-related gene expression against *Ostrinia furnacalis*	Downregulation of photosynthesis-related genes and reduced chlorophyll content; field-scale dose optimization remains necessary	[152]
NERICA 4 (*Oryza sativa* and *O. glaberrima*)	Hydro priming + priming liquid	Seed vigor maintained for up to 90 days of storage	Seed vigor and viability declined after 120 days owing to accelerated metabolic activity	[149]
*Lactuca sativa*	*Microbacterium* spp. (29 strains screened)	Three strains increased radicle biomass by approximately 45% in vitro	Strong strain specificity; effects mainly associated with volatile compounds	[153]
*Cicer arietinum*	Cyanobacterial consortium coating	Germination and vigor maintained for up to six months of storage	Progressive decline during prolonged storage; validation under multi-location field conditions is still needed	[154]
*Brassica napus*	*Pseudomonas fluorescens* dry biopriming	Kimchi paste markedly improved bacterial shelf-life; hydropriming enhanced germination uniformity	Biopriming alone reduced germination uniformity; response depended on salt concentration during priming	[155]
Multiple crops	Four-PGPR SynCom: *Azospirillum brasilense*, *Azotobacter chroococcum*, *P. fluorescens*, *B. subtilis*	Increased germination by 23% and significantly improved seedling vigor under saline conditions (14 dS m^−1^)	Functional interactions among consortium members require mechanistic investigation	[156]

PGPR: plant growth-promoting rhizobacteria.

## Data Availability

No new data were created or analyzed in this study. Data sharing is not applicable to this article.

## References

[1] Kermode A.R. (2018). Regulatory Mechanisms in the Transition from Seed Development to Germination: Interactions Between the Embryo and the Seed Environment. Seed Development and Germination.

[2] Boesewinkel F.D., Bouman F. (2018). The Seed: Structure and Function. Seed Development and Germination.

[3] Xu H., Wang F., Damari R.N., Chen X., Lin Z. (2024). Molecular mechanisms underlying the signal perception and transduction during seed germination. Mol. Breed..

[4] Seale M., Nakayama N. (2020). From passive to informed: Mechanical mechanisms of seed dispersal. New Phytol..

[5] Jatana B.S., Grover S., Ram H., Baath G.S. (2024). Seed priming: Molecular and physiological mechanisms underlying biotic and abiotic stress tolerance. Agronomy.

[6] Janah I., Elhasnaoui A., Abouloifa H., Ait-El-Mokhtar M., Ben Laouane R. (2025). Hormonal priming to increase germination of *Stevia rebaudiana* Bertoni seeds in saline environments. Int. J. Plant Biol..

[7] Janah I., Elhasnaoui A., Ben Laouane R., Ait-El-Mokhtar M., Anli M. (2025). Exploring seed priming as a strategy for enhancing abiotic stress tolerance in cereal crops. Stresses.

[8] Ashraf M., Foolad M.R. (2005). Pre-sowing seed treatment—A shotgun approach to improve germination, plant growth, and crop yield under saline and non-saline conditions. Adv. Agron..

[9] Zulfiqar F. (2021). Effect of seed priming on horticultural crops. Sci. Hortic..

[10] Rhaman M.S., Rauf F., Tania S.S., Khatun M. (2020). Seed priming methods: Application in field crops and future perspectives. Asian J. Res. Crop Sci..

[11] Paul S., Roychoudhury A. (2017). Seed priming with spermine and spermidine regulates the expression of diverse groups of abiotic stress-responsive genes during salinity stress in the seedlings of indica rice varieties. Plant Gene.

[12] Chen S., Liu H., Yangzong Z., Gardea-Torresdey J.L., White J.C., Zhao L. (2023). Seed priming with reactive oxygen species-generating nanoparticles enhances maize tolerance to multiple abiotic stresses. Environ. Sci. Technol..

[13] Yadav N., Bora S., Devi B., Upadhyay C., Singh P. (2024). Nanoparticle-mediated defense priming: A review of strategies for enhancing plant resilience against biotic and abiotic stresses. Plant Physiol. Biochem..

[14] Ali K., Mubasher H.M., Sher A., Sattar A., Manaf A. (2023). Seed priming for abiotic stress tolerance. Climate-Resilient Agriculture.

[15] Jisha K.C., Vijayakumari K., Puthur J.T. (2013). Seed priming for abiotic stress tolerance: An overview. Acta Physiol. Plant..

[16] Nair A.U., Bhukya D.P.N., Sunkar R., Chavali S., Allu A.D. (2022). Molecular basis of priming-induced acquired tolerance to multiple abiotic stresses in plants. J. Exp. Bot..

[17] Mulla S., Ahmed R., Singh K.K., Singh S.K., Deshmukh N., Inamdar F.K. (2023). Climate change effect on climate parameters like temperature, rainfall and water resources sectors in India. Climate Change and Water Security.

[18] Khan A.R., Ghosh U.K., Hossain S., Mahmud A., Rahman M., Biswas J.C. (2024). Agricultural abiotic stresses in the tropical and subtropical agroecosystem. Climate Change and Soil-Water-Plant Nexus: Agriculture and Environment.

[19] Shil S., Ashwath M.N., Das S., Vats P., Raj A.K., Dash U., Bhardwaj A. (2025). Seed biopriming: A sustainable solution for enhancing seed vigor and crop productivity. Advances in Seed Quality Evaluation and Improvement.

[20] Cardarelli M., Woo S.L., Rouphael Y., Colla G. (2022). Seed treatments with microorganisms can have a biostimulant effect by influencing germination and seedling growth of crops. Plants.

[21] Srivastava S., Tyagi R., Sharma S. (2024). Seed biopriming as a promising approach for stress tolerance and enhancement of crop productivity: A review. J. Sci. Food Agric..

[22] Mitra D., Mondal R., Khoshru B., Shadangi S., Das Mohapatra P.K., Panneerselvam P. (2021). Rhizobacteria-mediated seed biopriming triggers resistance and plant growth for sustainable crop production. Curr. Res. Microb. Sci..

[23] Yang P., Lu L., Condrich A., Muni G.A., Scranton S., Xu S., Xia Y., Huang S. (2025). Innovative approaches for engineering the seed microbiome to enhance crop performance. Seeds.

[24] Qu Z., Zhao H., Zhang H., Wang Q., Yao Y., Cheng J., Lin Y., Xie J., Fu Y., Jiang D. (2020). Bio-priming with a hypovirulent phytopathogenic fungus enhances the connection and strength of microbial interaction networks in rapeseed. npj Biofilms Microbiomes.

[25] Subašić M., Selović A., Dahija S., Demir A., Samardžić J., Bonomo A., Rigano G., Giosa D., Karalija E. (2026). Biopriming-induced transcriptomic memory enhances cadmium tolerance in the Cd hyperaccumulator *Silene sendtneri*. Plants.

[26] Fiodor A., Ajijah N., Dziewit L., Pranaw K. (2023). Biopriming of seed with plant growth-promoting bacteria for improved germination and seedling growth. Front. Microbiol..

[27] Upretee P., Bandara M.S., Tanino K.K. (2024). The role of seed characteristics on water uptake preceding germination. Seeds.

[28] Olofintila O.E., Noel Z.A. (2023). Soybean and cotton spermosphere soil microbiome shows dominance of soilborne copiotrophs. Microbiol. Spectr..

[29] Johnston-Monje D., Vergara L.I., Lopez-Mejia J., White J.F. (2023). Plant microbiomes as contributors to agricultural terroir. Front. Agron..

[30] Joubert O., Arnault G., Barret M., Simonin M. (2025). Sowing success: Ecological insights into seedling microbial colonisation for robust plant microbiota engineering. Trends Plant Sci..

[31] Hazra A., Das S. (2024). The molecular and metabolic events behind different germination stages of rice seeds: A metabolomics perspective. J. Sci. Food Agric..

[32] Nelson E.B. (2018). The seed microbiome: Origins, interactions, and impacts. Plant Soil.

[33] Martins S.J., Medeiros F.H.V., Lakshmanan V., Bais H.P. (2018). Impact of seed exudates on growth and biofilm formation of *Bacillus amyloliquefaciens* ALB629 in common bean. Front. Microbiol..

[34] Pal G., Kumar K., Verma A., Verma S.K. (2022). Seed-inhabiting bacterial endophytes of maize promote seedling establishment and provide protection against fungal disease. Microbiol. Res..

[35] Mohamed N.G., Mokhtar A., Khaled Y., Mohamed N., Mahmoud A. (2025). Seed biopriming with *Bacillus nematocida* enhances drought tolerance in maize via regulation of stress-responsive genes. Sci. Rep..

[36] Hadj Brahim A., Ben Ali M., Daoud L., Jlidi M., Akremi I., Hmani H., Feto N.A., Ben Ali M. (2022). Biopriming of durum wheat seeds with endophytic diazotrophic bacteria enhances tolerance to *Fusarium* head blight and salinity. Microorganisms.

[37] Yassin M.A., George N., Shabaan L., Gouda Y. (2024). Biopriming of maize with its endophyte *Aspergillus fumigatus* reinforces resistance to salinity stress and improves physiological traits. BMC Plant Biol..

[38] Tiwari P., Adil M., Park K.I. (2025). Biopriming with endophytes improves plant resilience toward climate-smart agriculture. World J. Microbiol. Biotechnol..

[39] Timofeeva A.M., Galyamova M.R., Sedykh S.E. (2023). Plant growth-promoting rhizobacteria: Nitrogen fixation, phosphate solubilization, siderophore production and other biological activities. Plants.

[40] Rawat L., Bisht T.S., Kukreti A. (2022). Potential of seed biopriming with *Trichoderma* in ameliorating salinity stress and providing resistance against leaf blast disease in finger millet (*Eleusine coracana* L.). Indian Phytopathol..

[41] Mustafa S., Kabir S., Shabbir U., Batool R. (2019). Plant growth-promoting rhizobacteria in sustainable agriculture: From theoretical to pragmatic approach. Symbiosis.

[42] Berg G., Kusstatscher P., Abdelfattah A., Cernava T., Smalla K. (2021). Microbiome modulation: Toward a better understanding of plant microbiome response to microbial inoculants. Front. Microbiol..

[43] Tripathi A.N., Meena B.R., Pandey K.K., Singh J. (2020). Microbial bioagents in agriculture: Current status and prospects. New Frontiers in Stress Management for Durable Agriculture.

[44] Bahrani A., Pourreza J., Joo M.H. (2010). Response of winter wheat to co-inoculation with *Azotobacter* and arbuscular mycorrhizal fungi (AMF) under different sources of nitrogen fertilizer. Am.-Eurasian J. Agric. Environ. Sci..

[45] Rozier C., Erban A., Kopka J., Hamzaoui J., Legendre L., Lemoine D., Czarnes S., Bertrand C., Nesme X. (2019). Biopriming of maize germination by the plant growth-promoting rhizobacterium *Azospirillum lipoferum* CRT1. J. Plant Physiol..

[46] Abbasi A., Mehri S., Solimanzadeh H., Alipour S. (2023). Growth, yield, and photosynthetic dry matter remobilization response of barley to plant growth-promoting rhizobacteria (PGPR) and nitrogen. Philipp. Agric. Sci..

[47] Mahdi I., Fahsi N., Hafidi M., Allaoui A., Biskri L. (2020). Plant growth enhancement using rhizospheric halotolerant phosphate-solubilizing bacterium *Bacillus licheniformis* QA1 and *Enterobacter asburiae* QF11 isolated from *Chenopodium quinoa*. Microorganisms.

[48] Sarić-Krsmanović M., Božić D., Radivojević L., Gajić-Umiljendić J., Šantrić L., Vrbničanin S. (2017). Effects of plant growth-promoting rhizobacteria (PGPR) and cover crops on seed germination and early establishment of field dodder (*Cuscuta campestris* Yunck.). Pestic. Fitomed..

[49] Hasanović M., Durmić-Pašić A., Karalija E. (2025). Enhancing nickel stress tolerance in Micro-Tom tomatoes through biopriming with Paraburkholderia phytofirmans PsJN: Insights into growth and physiological responses. Front. Microbiol..

[50] Roslan M.A.M., Zulkifli N.N., Sobri Z.M., Zuan A.T.K., Cheak S.C., Abdul Rahman N.A. (2020). Seed biopriming with P- and K-solubilizing *Enterobacter hormaechei* sp. improves the early vegetative growth and the P and K uptake of okra (*Abelmoschus esculentus*) seedlings. PLoS ONE.

[51] Villani A., Tommasi F., Paciolla C. (2021). The arbuscular mycorrhizal fungus *Glomus viscosum* improves tolerance to Verticillium wilt in artichoke by modulating the antioxidant defense system. Cells.

[52] Timofeeva A.M., Galyamova M.R., Sedykh S.E. (2024). How do plant growth-promoting bacteria use plant hormones to regulate stress reactions?. Plants.

[53] Tripathi R., Aravind T., Kumar S., Keswani C., Singh S.P., Tewari R., Tewari A.K., Singh K.P., Minkina T. (2025). Microbial phytohormones: The potential orchestrators of plant growth and defense. Discov. Plants.

[54] Aloo B.N., Dessureault-Rompré J., Tripathi V., Nyongesa B.O., Were B.A. (2023). Signaling and crosstalk of rhizobacterial and plant hormones that mediate abiotic stress tolerance in plants. Front. Microbiol..

[55] Curá J.A., Franz D.R., Filosofía J.E., Balestrasse K.B., Burgueño L.E. (2017). Inoculation with *Azospirillum* sp. and *Herbaspirillum* sp. increases maize tolerance to drought stress. Microorganisms.

[56] Vardharajula S., Ali S.Z., Grover M., Reddy G., Bandi V. (2011). Drought-tolerant plant growth-promoting *Bacillus* spp.: Effects on growth, osmolytes and antioxidant status of maize under drought stress. J. Plant Interact..

[57] Kang S.M., Radhakrishnan R., Khan A.L., Kim M.J., Park J.M., Kim B.R., Lee I.J. (2019). Integrated phytohormone production by the plant growth-promoting rhizobacterium Bacillus tequilensis SSB07 induces thermotolerance in soybean. J. Plant Interact..

[58] Romão I.R., Gomes J.C., Silva D., Vilchez J.I. (2025). The seed microbiota from an application perspective: An underexplored frontier in plant–microbe interactions. Crop Health.

[59] Saghafi D., Ghorbanpour M., Lajayer B.A. (2018). Efficiency of *Rhizobium* strains as plant growth-promoting rhizobacteria on morphophysiological properties of *Brassica napus* L. under salinity stress. J. Soil Sci. Plant Nutr..

[60] Ji J., Yuan D., Jin C., Wang G., Li X., Guan C. (2020). Enhancement of growth and salt tolerance of rice seedlings (*Oryza sativa* L.) by regulating ethylene production with a novel halotolerant PGPR strain *Glutamicibacter* sp. YD01 containing ACC deaminase activity. Acta Physiol. Plant..

[61] Haque M.M., Biswas M.S., Mosharaf M.K., Haque M.A., Islam M.S., Nahar K., Uddin M.K., Khatun M.T., Islam M.T., Islam M.M. (2022). Halotolerant biofilm-producing rhizobacteria mitigate seawater-induced salt stress and promote growth of tomato. Sci. Rep..

[62] Yasmin H., Naeem S., Bakhtawar M., Jabeen Z., Nosheen A., Naz R., Keyani R., Mumtaz S., Hassan M.N. (2020). Halotolerant rhizobacteria Pseudomonas pseudoalcaligenes and Bacillus subtilis mediate systemic tolerance in hydroponically grown soybean (*Glycine max* L.) against salinity stress. PLoS ONE.

[63] Šírová J., Sedlářová M., Piterková J., Luhová L., Petřivalský M. (2011). The role of nitric oxide in the germination of plant seeds and pollen. Plant Sci..

[64] Xu W., Miao Y., Kong J., Lindsey K., Zhang X., Min L. (2024). ROS signaling and its involvement in abiotic stress with emphasis on heat stress-driven anther sterility in plants. Crop Environ..

[65] El-Maarouf-Bouteau H., Bailly C. (2008). Oxidative signaling in seed germination and dormancy. Plant Signal. Behav..

[66] Thiruvengadam R., Venkidasamy B., Easwaran M., Chi H.Y., Thiruvengadam M., Kim S.H. (2024). Dynamic interplay of reactive oxygen and nitrogen species (ROS and RNS) in plant resilience: Unveiling the signaling pathways and metabolic responses to biotic and abiotic stresses. Planta.

[67] Johnson R., Puthur J.T. (2021). Seed priming as a cost-effective technique for developing plants with cross-tolerance to salinity stress. Plant Physiol. Biochem..

[68] Ali M., Kaderbek T., Khan M.A., Skalicky M., Brestic M., Elsabagh M., El Sabagh A. (2025). Biosynthesis and multifaceted roles of reactive species in plant defense mechanisms during environmental cues. Plant Stress.

[69] Khan M., Ali S., Al Azzawi T.N.I., Saqib S., Ullah F., Ayaz A., Zaman W. (2023). The key roles of ROS and RNS as signaling molecules in plant–microbe interactions. Antioxidants.

[70] El-Mougy N.S., Abdel-Kader M.M. (2008). Long-term activity of bio-priming seed treatment for biological control of faba bean root rot pathogens. Australas. Plant Pathol..

[71] Singh P., Vaishnav A., Liu H., Xiong C., Singh H.B., Singh B.K. (2023). Seed biopriming for sustainable agriculture and ecosystem restoration. Microb. Biotechnol..

[72] Pinedo I., Ledger T., Greve M., Poupin M.J. (2015). *Burkholderia phytofirmans* PsJN induces long-term metabolic and transcriptional changes involved in *Arabidopsis thaliana* salt tolerance. Front. Plant Sci..

[73] Cecere G. (2021). Small RNAs in epigenetic inheritance: From mechanisms to trait transmission. FEBS Lett..

[74] Wagh S.G., Patil A.M., Patil G.B., Mankar S.P., Rastogi K., Nishiguchi M. (2025). Small RNA and epigenetic control of plant immunity. DNA.

[75] Bharadwaj R., Noceda C., Mohanapriya G., Kumar S.R., Thiers K.L.L., Costa J.H., Kumari A., Grosskinsky D.K., Neumann G., Arnholdt-Schmitt B. (2021). Adaptive reprogramming during early seed germination requires temporarily enhanced fermentation: A critical role for alternative oxidase regulation that concerns also microbiota effectiveness. Front. Plant Sci..

[76] Lephatsi M.M., Meyer V., Piater L.A., Dubery I.A., Tugizimana F. (2021). Plant responses to abiotic stresses and rhizobacterial biostimulants: Metabolomics and epigenetics perspectives. Metabolites.

[77] Gagné-Bourque F., Mayer B.F., Charron J.-B., Vali H., Bertrand A., Jabaji S. (2015). Accelerated growth rate and increased drought stress resilience of the model grass *Brachypodium distachyon* colonized by *Bacillus subtilis* B26. PLoS ONE.

[78] Louis N., Dhankher O.P., Puthur J.T. (2023). Seed priming can enhance and retain stress tolerance in ensuing generations by inducing epigenetic changes and transgenerational memory. Physiol. Plant..

[79] Chopra S., Sharma S.G., Kaur S., Kumar V., Guleria P. (2025). Understanding the microRNA-mediated regulation of plant–microbe interaction and its role in abiotic and biotic stress tolerance in plants. Physiol. Mol. Plant Pathol..

[80] Liu J., Lu Y., Chen X., Liu X., Gu Y., Li F. (2025). The silent conversation: How small RNAs shape plant–microbe relationships. Int. J. Mol. Sci..

[81] Dzinyela R., Alhassan A.R., Kiani-Pouya A., Rasouli F., Yang L., Movahedi A. (2023). Role of small RNAs in plant stress response and their potential to improve crops. Crop Pasture Sci..

[82] Ravet A., Zervudacki J., Singla-Rastogi M., Charvin M., Thiebeauld O., Perez-Quintero A.L., Courgeon L., Candat A., Lebeau L., Fortunato A.E. (2025). Vesicular and non-vesicular extracellular small RNAs direct gene silencing in a plant-interacting bacterium. Nat. Commun..

[83] Wang W., Zhang F., Cui J., Chen D., Liu Z., Hou J., Zhang R., Liu T. (2021). Identification of microRNA-like RNAs from *Trichoderma asperellum* DQ-1 during its interaction with tomato roots using bioinformatic analysis and high-throughput sequencing. PLoS ONE.

[84] Crisp P.A., Ganguly D., Eichten S.R., Borevitz J.O., Pogson B.J. (2016). Reconsidering plant memory: Intersections between stress recovery, RNA turnover, and epigenetics. Sci. Adv..

[85] De Palma M., Grillo S., Massarelli I., Costa A., Baloglu M.C., Vitiello A., Lorito M., Woo S.L., Scala F., Ercolano M.R. (2019). Transcriptome reprogramming, epigenetic modifications and alternative splicing orchestrate the tomato root response to the beneficial fungus *Trichoderma harzianum*. Hortic. Res..

[86] Tiwari M., Devi B., Sinha S., Yadav N., Singh P. (2024). Intergenerational priming by *Trichoderma* alleviates drought stress in barley. Environ. Exp. Bot..

[87] Kapazoglou A., Drosou V., Argiriou A., Tsaftaris A.S. (2013). The study of a barley epigenetic regulator, *HvDME*, in seed development and under drought stress. BMC Plant Biol..

[88] Zhu H., Xie W., Xu D., Miki D., Tang K., Huang C.-F., Zhu J.-K. (2018). DNA demethylase ROS1 negatively regulates the imprinting of DOGL4 and seed dormancy in *Arabidopsis thaliana*. Proc. Natl. Acad. Sci. USA.

[89] Kim J.S., Lim J.Y., Shin H., Kim B.G., Yoo S.D., Kim W.T., Huh J.H. (2019). ROS1-dependent DNA demethylation is required for ABA-inducible NIC3 expression. Plant Physiol..

[90] Villagómez-Aranda A.L., García-Ortega L.F., Torres-Pacheco I., Guevara-González R.G. (2021). Whole-genome DNA methylation analysis in hydrogen peroxide overproducing transgenic tobacco resistant to biotic and abiotic stresses. Plants.

[91] Kadota Y., Shirasu K., Zipfel C. (2015). Regulation of the NADPH oxidase RBOHD during plant immunity. Plant Cell Physiol..

[92] Kang W., Zhu X., Wang Y., Chen L., Duan Y. (2018). Transcriptomic and metabolomic analyses reveal that bacteria promote plant defense during infection of soybean cyst nematode in soybean. BMC Plant Biol..

[93] Aydinoglu F., Kahriman T.Y., Balci H. (2023). Seed bio-priming enhanced salt stress tolerance of Zea mays L. seedlings by regulating the antioxidant system and miRNA expression. 3 Biotech.

[94] Panchal K., Sudhir A., Prajapati A.S. (2026). Engineering the plant microbiome: Synthetic community approaches to enhance crop protection. Front. Plant Sci..

[95] Sammauria R., Kumawat S., Kumawat P., Singh J., Jatwa T.K. (2020). Microbial inoculants: Potential tools for sustainability of agricultural production systems. Arch. Microbiol..

[96] Tariq A., Guo S., Farhat F., Shen X. (2025). Engineering synthetic microbial communities: Diversity and applications in soil for plant resilience. Agronomy.

[97] Jan S., Ashraf Bhat M., Kumar A., Altaf Wani M., Ahmad Bhat F., Kanth R.H., Shikari A.B., Bano H., Manzoor T., Altaf H. (2025). Plants response to different abiotic stresses. J. Crop Health.

[98] Akram W., Waqar S., Hanif S., Anjum T., Aftab Z.E.H., Li G., Saleem M., Umer M. (2024). Comparative effect of seed coating and biopriming of Bacillus aryabhattai Z-48 on seedling growth, growth promotion, and suppression of Fusarium wilt disease of tomato plants. Microorganisms.

[99] Shukla N., Awasthi R.P., Rawat L., Kumar J. (2015). Seed biopriming with drought-tolerant isolates of *Trichoderma harzianum* promotes growth and drought tolerance in *Triticum aestivum*. Ann. Appl. Biol..

[100] Martins S.J., Pasche J., Silva H.A.O., Selten G., Savastano N., Abreu L.M., Bais H.P., Garrett K.A., Kraisitudomsook N., Pieterse C.M. (2023). The use of synthetic microbial communities to improve plant health. Phytopathology.

[101] Wang Z., Wang S., He Q., Yang X., Zhao B., Zhang H., Deng Y. (2025). Ecological design of high-performance synthetic microbial communities: From theoretical foundations to functional optimization. ISME Commun..

[102] Kaur S., Egidi E., Qiu Z., Macdonald C.A., Verma J.P., Trivedi P., Wang J., Liu H., Singh B.K. (2022). Synthetic community improves crop performance and alters rhizosphere microbial communities. J. Sustain. Agric. Environ..

[103] Yin C., Hagerty C.H., Paulitz T.C. (2022). Synthetic microbial consortia derived from rhizosphere soil protect wheat against a soilborne fungal pathogen. Front. Microbiol..

[104] Flores-Duarte N.J., Navarro-Torre S., Mateos-Naranjo E., Redondo-Gómez S., Pajuelo E., Rodríguez-Llorente I.D. (2023). Nodule synthetic bacterial community as legume biofertilizer under abiotic stress in estuarine soils. Plants.

[105] Sulastri S., Rahmawati R.S., Roswanjaya Y.P., Romadhona E.I., Maretta D., Sukmadi R.B., Asiani N., Cindra Irawati A.F., Saryanah N.A. (2026). Root colonization by synthetic bacterial communities improves below- and above-ground traits in maize under combined abiotic stress. Ecol. Front..

[106] Khan M.Y., Nadeem S.M., Sohaib M., Waqas M.R., Alotaibi F., Ali L., Zahir Z.A., Al-Barakah F.N. (2022). Potential of plant growth-promoting bacterial consortium for improving the growth and yield of wheat under saline conditions. Front. Microbiol..

[107] Sathya A., Rehman V., Srinivas V., Kudapa H., Gopalakrishnan S. (2024). Efficacy of two different microbial consortia on salinity tolerance in chickpea: An *in planta* evaluation of biochemical, histochemical, and genomic responses. 3 Biotech.

[108] Alghamdy A., Rawat A., Parween S., Nagarajan A.P., Saad M.M., Hirt H. (2024). Mangrove endophytes shorten the life cycle of rice while enhancing yield and salt tolerance. bioRxiv.

[109] Yadav A., Chen M., Acharya S.M., Kim G., Yang Y., Zhao T.Z., Tsang E., Chakraborty R. (2025). A stable 15-member bacterial SynCom promotes *Brachypodium* growth under drought stress. Front. Microbiol..

[110] Herath Dissanayakalage S.S., Kaur J., Li T., Dimech A.M., Sawbridge T.I. (2025). Seed-derived synthetic microbial communities (SynComs) from *Medicago* wild relatives modulate early plant microbiome assembly and phenotypic traits in lucerne (*Medicago sativa* L.). Microorganisms.

[111] Zeyad M.T., Tiwari P., Ansari W.A., Kumar S.C., Kumar M., Chakdar H., Srivastava A.K., Singh U.B., Saxena A.K. (2022). Bio-priming with a consortium of *Streptomyces araujoniae* strains modulates defense responses in chickpea against *Fusarium* wilt. Front. Microbiol..

[112] Zaman W., Khalil A.A.K., Amin A., Ali S. (2025). Nanofabrication techniques for enhancing plant–microbe interactions in sustainable agriculture. Nanomaterials.

[113] Chen J., Zeng X., Yang W., Xie H., Ashraf U., Mo Z., Liu J., Li G., Li W. (2021). Seed priming with multiwall carbon nanotubes (MWCNTs) modulates seed germination and early growth of maize under cadmium (Cd) toxicity. J. Soil Sci. Plant Nutr..

[114] Soni A.T., Rookes J.E., Arya S.S. (2023). Chitosan nanoparticles as seed priming agents to alleviate salinity stress in rice (*Oryza sativa* L.) seedlings. Polysaccharides.

[115] Behl K., Jaiswal P., Pabbi S. (2024). Recent advances in microbial and nanoformulations for effective delivery and agricultural sustainability. Biocatal. Agric. Biotechnol..

[116] Wang X., He M., Wang X., Liu S., Luo L., Zeng Q., Wu Y., Zeng Y., Yang Z., Sheng G. (2024). Emerging nanochitosan for sustainable agriculture. Int. J. Mol. Sci..

[117] Sarver E., González-Morelo K.J., Christensen K.G., Lefevers H.M., Corbin K.R. (2025). Phyllosphere synthetic microbial communities: A new frontier in plant protection. BMC Plant Biol..

[118] Thirumurugan N.K., Velu G., Murugaiyan S., Maduraimuthu D., Ponnuraj S., Sharmila D.J., Subramanian K.S. (2025). Nano-biofertilizers: Utilizing nanopolymers as coating matrix—A comprehensive review. Environ. Res. Commun..

[119] Panichikkal J., Krishnankutty R.E. (2022). Chitosan and gold nanoparticles supplementation for augmentation of indole-3-acetic acid production by rhizospheric *Pseudomonas aeruginosa* and plant growth enhancement. Curr. Microbiol..

[120] Ravichandran M., Samiappan S.C., Rangaraj S., Murugan K., Al-Dhabi N.A., Karuppiah P. (2022). Nanoemulsion formulations with plant growth-promoting rhizobacteria for sustainable agriculture. Bio-Based Nanoemulsions for Agri-Food Applications.

[121] Karunakaran A., Fathima Y., Singh P., Beniwal R., Singh J., Ramakrishna W. (2024). Next-generation biofertilizers: Nanoparticle-coated plant growth-promoting bacteria biofertilizers for enhancing nutrient uptake and wheat growth. Agriculture.

[122] Abdulraheem M.I., Naz I., Pérez-Alvarez M., Hu J., Cadenas-Pliego G., Fawole O.A. (2026). Nanoparticle-induced cross-tolerance: A review of mechanisms for concurrent biotic and abiotic stress mitigation in crops. Plants.

[123] Singh K., Malla M.A., Kumar A., Yadav S. (2025). Toxicological concerns of nanomaterials in agroecosystems: Risk, fate, and analytical and regulatory assessment. Environ. Qual. Manag..

[124] Nikitha J., Alagu M. (2025). Post-genomic era in crop breeding. Plant Breeding 2050.

[125] Xu Y., Fu X. (2022). Reprogramming of plant central metabolism in response to abiotic stresses: A metabolomics view. Int. J. Mol. Sci..

[126] Dettmer K., Aronov P.A., Hammock B.D. (2007). Mass spectrometry-based metabolomics. Mass Spectrom. Rev..

[127] Mashabela M.D., Tugizimana F., Steenkamp P.A., Piater L.A., Dubery I.A., Mhlongo M.I. (2022). Untargeted metabolite profiling to elucidate rhizosphere and leaf metabolome changes of wheat cultivars (*Triticum aestivum* L.) treated with the plant growth-promoting rhizobacteria *Paenibacillus alvei* (T22) and *Bacillus subtilis*. Front. Microbiol..

[128] Rawat L., Singh Y., Shukla N., Kumar J. (2011). Alleviation of the adverse effects of salinity stress in wheat (*Triticum aestivum* L.) by seed biopriming with salinity-tolerant isolates of *Trichoderma harzianum*. Plant Soil.

[129] Chakraborti S., Bera K., Sadhukhan S., Dutta P. (2022). Bio-priming of seeds: Plant stress management and its underlying cellular, biochemical, and molecular mechanisms. Plant Stress.

[130] Abideen Z., Cardinale M., Zulfiqar F., Koyro H.W., Rasool S.G., Hessini K., Darbali W., Zhao F., Siddique K.H. (2022). Seed endophyte bacteria enhance drought stress tolerance in *Hordeum vulgare* by regulating physiological characteristics, antioxidant capacity, and mineral uptake. Front. Plant Sci..

[131] Ali B., Wang X., Saleem M.H., Sumaira, Hafeez A., Afridi M.S., Khan S., Zaib-Un-Nisa, Ullah I., Amaral Júnior A.T.d. (2022). PGPR-mediated salt tolerance in maize by modulating plant physiology, antioxidant defense, compatible solute accumulation, and biosurfactant-producing genes. Plants.

[132] Mahmoud A.M., Khalaf M.H., Reyad A.M., Korany S.M., Alsherif E.A., Shaghaleh H., Alhaj Hamoud Y., Sheteiwy M.S., El-Keblawy A., Ulhassan Z. (2025). Sustainable enhancement of basil quality and resilience through biopriming with *Pseudomonas* JP0825. BMC Plant Biol..

[133] Mona S.A., Hashem A., Abd_Allah E.F., Alqarawi A.A., Soliman D.W.K., Wirth S., Egamberdieva D. (2017). Increased drought resistance following *Trichoderma harzianum* treatment correlates with enhanced secondary metabolite and proline accumulation. J. Integr. Agric..

[134] Khaledi N., Dehshiri A., Hassani F. (2021). Effects of seed biopriming with *Trichoderma harzianum* on secondary metabolite production in cumin (*Cuminum cyminum* L.). Iran. J. Med. Aromat. Plants Res..

[135] Liu K., Deng F., Zeng F., Chen Z.H., Qin Y., Chen G. (2025). Plant growth-promoting rhizobacteria improve drought tolerance of crops: A review. Plant Growth Regul..

[136] Zhang H., Murzello C., Sun Y., Kim M.S., Xie X., Jeter R.M., Zak J.C., Dowd S.E., Paré P.W. (2010). Choline and osmotic stress tolerance induced in *Arabidopsis* by the soil bacterium *Bacillus subtilis* GB03. Mol. Plant-Microbe Interact..

[137] Bhaskaran M., Kokila M., Jagadeeshselvam N., Rahman H., Senthil N. (2017). Proteomic analysis of growth-promoting effects of biopriming in rice. Int. J. Curr. Microbiol. Appl. Sci..

[138] Puranik S., Mekali J., Damodaram K.J.P. (2025). Seed biopriming from basics to omics: Relieving plants from biotic stress through the microbial way. J. Basic Microbiol..

[139] Tong Z., Tao Z., Li F., He J., Qin S. (2026). Multi-omics integration reveals temporal partitioning between metabolic priming and proliferative expansion in PGPR-treated cherry plants. Int. J. Mol. Sci..

[140] Hillebrand H., Gurevitch J. (2014). Meta-analysis results are unlikely to be biased by differences in variance and replication between ecological laboratory and field studies. Oikos.

[141] Calisi R.M., Bentley G.E. (2009). Lab and field experiments: Are they the same animal?. Horm. Behav..

[142] Ben-Jabeur M., Kthiri Z., Djébali N., Karmous C., Hamada W. (2023). A case study of seed biopriming and chemical priming: Seed coating with two types of bioactive compounds improves the physiological state of germinating seeds in cereals. Cereal Res. Commun..

[143] Andersen S., Harrison G.W., Lau M.I., Rutström E.E. (2010). Preference heterogeneity in experiments: Comparing the field and laboratory. J. Econ. Behav. Organ..

[144] Prasad S.R., Kamble U.R., Sripathy K.V., Bhaskar K.U., Singh D.P., Singh D.P., Singh H.B., Prabha R. (2016). Seed bio-priming for biotic and abiotic stress management. Microbial Inoculants in Sustainable Agricultural Productivity.

[145] Monajjem S., Soltani E., Zainali E., Esfahani M., Ghaderi-Far F., Chaleshtori M.H., Rezaei A. (2023). Seed priming improves enzymatic and biochemical performances of rice during seed germination under low and high temperatures. Rice Sci..

[146] Wang W., He A., Peng S., Huang J., Cui K., Nie L. (2018). The effect of storage condition and duration on the deterioration of primed rice seeds. Front. Plant Sci..

[147] Gong M., Zheng M., Li X., Li Y., Qiao Z., Ren Y., Lv G. (2025). A microbial microencapsulation design of seed coating technology to boost wheat seed performance in saline soil. Chem. Biol. Technol. Agric..

[148] Rakshit A., Singh H.B. (2018). Advances in Seed Priming.

[149] Bore E.K., Ishikawa E., Libron J.A.M.A., Goto K., Odama E., Nakao Y., Yabuta S., Sakagami J.I. (2023). Primed seeds of NERICA 4 stored for long periods under high temperature and humidity conditions maintain germination rates. Appl. Sci..

[150] Mhada M., Zvinavashe A.T., Hazzoumi Z., Zeroual Y., Marelli B., Kouisni L. (2021). Bioformulation of silk-based coating to preserve and deliver *Rhizobium tropici* to *Phaseolus vulgaris* under saline environments. Front. Plant Sci..

[151] Chin J.M., Lim Y.Y., Ting A.S.Y. (2021). Biopolymers for biopriming of *Brassica rapa* seeds: A study on coating efficacy, bioagent viability and seed germination. J. Saudi Soc. Agric. Sci..

[152] Batool R., Umer M.J., Shabbir M.Z., Wang Y., Ahmed M.A., Guo J., Iqbal M., Ali S., Wang Z. (2022). Seed myco-priming improves crop yield and herbivory-induced defenses in maize by coordinating antioxidants and the jasmonic acid pathway. BMC Plant Biol..

[153] Suarez-Estrella F., Jurado M.M., Lopez-Gonzalez J.A., Toribio A., Martinez-Gallardo M.R., Estrella-Gonzalez M.J., Lopez M.J. (2023). Seed priming by application of *Microbacterium* spp. strains for control of *Botrytis cinerea* and growth promotion of lettuce plants. Sci. Hortic..

[154] Khatun M., Prasanna R., Bhardwaj A., Makur S., Lal S.K., Basu S., Kumar P.R. (2024). Developing microbial seed coating for enhancing seed vigour and prolonging storability in chickpea. S. Afr. J. Bot..

[155] Mitchener B., King J., Peters C., Peereboom A., Dobrowolska-Haywood M., Steinbrecher T., Leubner-Metzger G. (2025). Seed biopriming and long-term air-dry storage effects on *Pseudomonas fluorescens* viability and *Brassica napus* germination. Seed Sci. Res..

[156] Patel R.K. (2025). Biopriming of seeds with microbial consortia to enhance germination and early growth performance under saline stress conditions. Int. J. Plant Pathol. Microbiol..

[157] Sokra I., Somaly S., Meta H. (2026). CRISPR–Cas9-based genome editing in microbial biotechnology: Advances in metabolic engineering, fermentation systems, and industrial applications. J. Agric. Technol..

[158] dos Reis G.A., Martínez-Burgos W.J., Pozzan R., Pastrana Puche Y., Ocán-Torres D., de Queiroz Fonseca Mota P., Rodrigues C., Lima Serra J., Scapini T., Karp S.G. (2024). Comprehensive review of microbial inoculants: Agricultural applications, technology trends in patents, and regulatory frameworks. Sustainability.

[159] Ahmad A., Munawar N., Khan Z., Qusmani A.T., Khan S.H., Jamil A., Ashraf S., Ghouri M.Z., Aslam S., Mubarik M.S. (2021). An outlook on global regulatory landscape for genome-edited crops. Int. J. Mol. Sci..

[160] Dima O., Custers R., De Veirman L., Inzé D. (2023). EU legal proposal for genome-edited crops hints at a science-based approach. Trends Plant Sci..

[161] Mmbando G.S. (2023). The legal aspect of the current use of genetically modified organisms in Kenya, Tanzania, and Uganda. GM Crops Food.

[162] Jarrar H., El-Keblawy A., Albawab M., Ghenai C., Sheteiwy M. (2024). Seed priming as a promising technique for sustainable restoration of drylands. Restor. Ecol..

[163] Liakos K.G., Busato P., Moshou D., Pearson S., Bochtis D. (2018). Machine Learning in Agriculture: A Review. Sensors.

[164] van Klompenburg T., Kassahun A., Catal C. (2020). Crop Yield Prediction Using Machine Learning: A Systematic Literature Review. Comput. Electron. Agric..

[165] Papoutsoglou G., Tarazona S., Lopes M.B., Klammsteiner T., Ibrahimi E., Eckenberger J., Novielli P., Tonda A., Simeon A., Shigdel R. (2023). Machine learning approaches in microbiome research: Challenges and best practices. Front. Microbiol..

[166] Ghanbari M., Nasrabadi M. (2026). An integrated artificial intelligence and nano-biostimulant seed coating system enhances seed germination and seedling growth under drought stress. Res. Sq..

[167] Fouad N., Elzayat E.M., Amr D., El-Khishin D.A., Radwan K.H., Youssef A., Khalaf A.A., Ahmed H.A., Radwan E.H., Tawkaz S. (2026). Rhizosphere microbiome engineering for climate-smart agriculture: From synthetic consortia to precision decision support. Microorganisms.

[168] Volke D.C., Orsi E., Nikel P.I. (2023). Emergent CRISPR–Cas-based technologies for engineering non-model bacteria. Curr. Opin. Microbiol..

[169] Shahid M., Singh U.B., Khan M.S., Singh P., Kumar R., Singh R.N., Kumar A., Singh H.V. (2023). Bacterial ACC deaminase: Insights into enzymology, biochemistry, genetics, and potential role in amelioration of environmental stress in crop plants. Front. Microbiol..

[170] Puja H., Mislin G.L.A., Rigouin C. (2023). Engineering siderophore biosynthesis and regulation pathways to increase diversity and availability. Biomolecules.

